# Revisiting the Social Contract: Physicians as Community Health Promoters

**Published:** 2007-06-15

**Authors:** Margaret Gadon

**Affiliations:** Health Disparities, American Medical Association, Professional Standards

The Shaw family is a typical U.S. family whose members live in a suburb of a small midwestern city. Mr and Mrs Shaw have three school-aged children, work full time, and earn a combined income of $75,000. They commute to work and drive an additional seven hours per week for errands and social and school events. On average, they eat out twice each week, and on a third night Mrs Shaw picks up some prepared food from the supermarket for dinner. Both adults work out at the local health club on weekends. Mr and Mrs Shaw, who are in their early 40s, are both overweight but have no other overt medical problems. One of the children has moderate asthma that is kept under control with the support of the school nurse. They visit their physicians regularly and attempt to follow all health advice. Physicians for the Shaw family have provided its members with detailed information on maintaining optimum health and changing unhealthy behaviors.

Are these physicians doing all they can to promote the health of the Shaw family? Traditionalists would most likely say yes. However, proponents of a greater professional role for physicians would not agree. They believe that the role of physicians extends further, to include a commitment to exert their influence on the many social, economic, and environmental factors that cause disease. This social contract between physicians and patients has been well described and justified by various leaders in medicine (1-7). In their 2004 paper, "Physician–Citizens — Public Roles and Professional Obligations," Gruen et al argue that social accountability is inherent to the profession of physician (7). They propose a model of physician responsibility that correlates the expected level of engagement in socioeconomic domains with the degree to which these factors adversely affect individual health (7).

Justifiably, physicians continue to be viewed as credible leaders within their communities. This privileged position provides them with the power to leverage public opinion in domains other than the strictly medical. Clear evidence exists that a physician's recommendation to stop smoking cigarettes is one of the most effective factors in promoting smoking cessation (8). Such efforts in the areas of injury prevention, environmental health, and nutrition that extend to a broader societal level are likely to be equally effective.

Physicians can promote health outside the traditional medical venue in multiple ways. One strategy is for physicians to work with local business networks to ensure that healthful foods are available throughout the community. Another is to appeal directly to municipal authorities for community recreational facilities such as parks and walking trails. Physicians can also promote community health by participating in local school programs designed to prevent obesity and school violence and by encouraging local, state, and national professional societies to advocate for legislative and policy changes to improve physical and social conditions that adversely affect people's health.

In this issue of *Preventing Chronic Disease,* Navarro et al discuss the recommendations of a national panel of experts to guide future activities related to community health at the Centers for Disease Control and Prevention's National Center for Chronic Disease Prevention and Health Promotion (9). The eight recommendations described in the report cover policy change, workforce issues, surveillance activities, and increased federal resources dedicated to community health promotion. Physicians working in concert with the public health system have the potential to develop collaborations that will enhance the health of their patients and that of the communities in which they live.

Yet how can we expect overburdened physicians to incorporate these added responsibilities into their daily practices of medicine? A first step could be a reassessment of their current system of practice and possible redesign of their offices to provide care more efficiently, equally, and effectively. Physicians might also work with their local medical societies and public health departments to identify public health and community resources that can complement and reinforce patient care. They can encourage patients to use these resources. Ultimately, working in partnership with community organizations could allow physicians to maintain continuity of care without overtaxing their office practices. As quality of care becomes increasingly incentivized, physicians can turn to community partnering as a means of achieving both optimum quality of care and healthier lifestyles for their patients. For the Shaw family, this could mean the difference between being at risk for and free from disease.
